# On fair, effective and efficient REDD mechanism design

**DOI:** 10.1186/1750-0680-4-11

**Published:** 2009-11-27

**Authors:** Michael Obersteiner, Michael Huettner, Florian Kraxner, Ian McCallum, Kentaro Aoki, Hannes Böttcher, Steffen Fritz, Mykola Gusti, Petr Havlik, Georg Kindermann, Ewald Rametsteiner, Belinda Reyers

**Affiliations:** 1 International Institute for Applied Systems Analysis, Schlossplatz 1, Laxenburg, Austria; 2 Max-Planck-Institute for Biogeochemistry, Hans-Knoll-Str 10, 07745 Jena, Germany; 3 Friedrich-Schiller-University Jena, GSBC-EIC 'The Economics of Innovative Change', Carl-Zeiss-Str 3, 07743 Jena, Germany; 4 The Council for Scientific and Industrial Research, Meiring Naudé Road, Brummeria, Pretoria, South Africa

## Abstract

The issues surrounding 'Reduced Emissions from Deforestation and Forest Degradation' (REDD) have become a major component of continuing negotiations under the United Nations Framework Convention on Climate Change (UNFCCC). This paper aims to address two key requirements of any potential REDD mechanism: first, the generation of measurable, reportable and verifiable (MRV) REDD credits; and secondly, the sustainable and efficient provision of emission reductions under a robust financing regime.

To ensure the supply of MRV credits, we advocate the establishment of an 'International Emission Reference Scenario Coordination Centre' (IERSCC). The IERSCC would act as a global clearing house for harmonized data to be used in implementing reference level methodologies. It would be tasked with the collection, reporting and subsequent processing of earth observation, deforestation- and degradation driver information in a globally consistent manner. The IERSCC would also assist, coordinate and supervise the computation of national reference scenarios according to rules negotiated under the UNFCCC. To overcome the threats of "market flooding" on the one hand and insufficient economic incentives for REDD on the other hand, we suggest an 'International Investment Reserve' (IIR) as REDD financing framework. In order to distribute the resources of the IIR we propose adopting an auctioning mechanism.

Auctioning not only reveals the true emission reduction costs, but might also allow for incentivizing the protection of biodiversity and socio-economic values. The introduced concepts will be vital to ensure robustness, environmental integrity and economic efficiency of the future REDD mechanism.

## Introduction

### The REDD process and the need for observations and decision support

Post-2012 emission mitigation strategies must lead to drastic emission reductions of greenhouse gases (GHGs) to prevent dangerous climate change. Accounting for some 18 percent of global anthropogenic GHG emissions in 2004 the reduction of emissions from deforestation and forest degradation (REDD) has become a prominent potential mitigation wedge. In effect, demonstration activities have flourished since the mandate given in the Bali Road Map of 2007 (UNFCCC Decision 2/CMP.13). Initiatives include the World Bank-hosted Forest Carbon Partnership Facility and Forest investment program, the UN-REDD program, Norway's International Climate and Forest Initiative, Australia's International Forest Carbon Initiative, and many other bilateral and private programs and projects. About 40 developing countries have already engaged in the process of designing REDD strategies. At times these various initiatives struggle to identify their synergies and avoid confusion regarding methodological and technical challenges. In particular, any system generating REDD credits is likely to operate within the scope of the principles stated in the Poznan Ministerial statement. These include, inter alia, the development of transparent, collaborative, balanced and inclusive international arrangements to support national REDD efforts. Decision makers also stressed that a reliable framework for measuring, reporting and verification (MRV) is crucial to the integrity and credibility of REDD. Key to the supply of MRV REDD credits is robust and consistent greenhouse gas (GHG) observation and monitoring systems combined with sound accounting methodologies and appropriate reference emission scenarios of deforestation and forest degradation (DD).

With respect to GHG accounting much progress has been achieved so far. It is generally believed that cost effective systems for estimating and monitoring deforestation and changes in carbon stocks can be designed and implemented using a combination of remote sensing assessments and ground based measurements [1]. However, guidance is needed to ensure comparable estimates when remote sensing is used, along with access to data, know-how and capacity building. Addressing forest degradation is especially difficult in this regard, but knowing the causes of degradation can help in designing meaningful stratified sampling approaches to measure it.

In short, the observation and monitoring challenges should not be viewed as a stumbling block for REDD policies to go ahead. However, efforts must be coordinated and streamlined through a robust international institutional arrangement, otherwise the environmental integrity and economic effectiveness of REDD is at risk.

### Challenges in the design of reference levels and financial compensation

One of the most challenging aspects in designing a REDD mechanism is the estimation of reference levels (RL). They describe the amount of net/gross emissions and removals from a geographical area under a business-as-usual (BAU) development path. By describing the future emission pathway without any climate protection measures, reference scenarios are crucial to determine the success of emission reduction performances. Reference level can be solely based on historical emission trajectories or additionally take into account circumstances such as global deforestation rate, national forest area or deforestation drivers [2,3].

When it comes to setting RL there is less clarity on an agreeable methodology. This is related to different country circumstances and interests. On the one hand, countries with low past deforestation rates and potential high future deforestation will not agree to purely historically derived RL. To benefit from REDD and to prevent future deforestation those parties will rather propose to consider 'national circumstances' for RL setting, e.g. through a so-called 'Development Adjustment Factor'. On the other hand, many developing countries still lack sufficient technical and expert capacity to develop proper RL methodologies. Furthermore, if the process of developing and reporting such RL is not carefully designed, there is a risk of creating a so-called "lemons market" [4]. This occurs when the seller knows considerably more about the real quality of a product than the buyer, resulting in a reduced quality of supply of the respective product. In a REDD context, the "lemons" would materialize in the form of globally inconsistent and inflated RL adjustments, leading to non-additional emission reductions. Under a carbon market scheme this potentially results in an oversupply of cheap REDD credits. Because negative emission deviation from the RL would be matched by financial compensation, a credible method for the measurement of additional REDD units is absolutely essential for financial efficiency in the light of scarce resources dedicated to REDD [5] and avoiding the risk of artificial RL inflation [6].

Besides RL design the choice of the financial mechanism for a future REDD regime is intensely debated. Again, this is partly related to different developing country circumstances, but here the discussion is dominated by concerns about managing the potential oversupply for REDD credits under a market or lacking demand under a fund mechanism. Another major concern is the negative socio-economic and environmental effect of a sole carbon focus for REDD [7]. To overcome these risks the REDD mechanism might distribute compensation benefits (e.g. credits) not only based on the amount of emissions reduced, but also on the ecological and social value of the forests in question. Ideally, such form of REDD mechanism would aim to distribute credits to those REDD activities that provide the maximum total benefit from emission reduction, ecological and social values. However, since the financial consideration of co-benefits is currently only favoured by some parties its compulsory inclusion is likely to overburden the REDD policy negotiations. Additionally, financing and monitoring challenges previously mentioned also apply to co-benefit valuation.

This paper describes two linked proposals for the essential building blocks of REDD policy implementation. First, we propose an institutional REDD design aimed at generating globally consistent reference scenarios at the country level from which to derive MRV REDD credits. The second part of the paper describes a possible financial mechanism design for generating such REDD credits in an economically efficient manner, while incentivizing the valuation of forest co-benefits.

## Discussion

### Institutional support for transparent, fair and efficient reference level setting

Globally consistent DD emission reference scenarios at the national level are important for a large number of reasons. These include avoiding international leakage as well as ensuring transparency, fairness and efficiency. Fairness relates to the issue of relative distributional gain of financial resources made available. Compensation of future REDD actions against a historical RL will favour countries with a high historical emissions on a relative scale. This will increase the risk that future drivers of deforestation geographically shift to historical low deforestation countries and, thus, create asymmetric winner/looser profiles between REDD countries. In this sense low deforestation countries loose out two times under a purely historical baseline setting. First their supply potential for REDD is decreased and secondly their true baseline will be pushed up due to international leakage of REDD actions implemented in high deforestation countries. On a total market level "over-compensation" by countries with historically high deforestation due to a grandfathering rule will compromise both environmental integrity and cost effectiveness of REDD. Finally, such 'over-compensation' could lead to the supply of non-additional emission reductions and thus to an inflation of REDD credits [8].

Irrespective of the fact that reliable historical DD data do not exist for the Pan-tropical belt, the currently proposed methods to quantify RL on historical information will be insufficient without the consideration of national circumstances (drivers) and global data streamlining. Thus, we propose a system of establishing reliable and acceptable RLs based on a global forest information coordination body and RL algorithm implementation centre.

### Data and quality requirements for reference level determination

The determination of the 'true' RL will not only shape global efficiency, but also be an important component for countries' planning REDD actions - regardless of how emissions reduction will be credited for. It is important to note that the 'true' BAU scenario does not have to be the same as the crediting RL [9]. The latter can be influenced by the 'Development Adjustment Factor' or eventually be the outcome of a negotiated "formula". Pure reliance on negotiation, however, potentially leads to political bargaining by strong actors. This could disadvantage less powerful developing countries in gaining financial access to REDD resources and threaten the environmental effectiveness of the REDD mechanism.

In the interest of fairness and efficiency, the final aim for RL determination will be that the 'true' BAU scenario and the crediting RL converge or in cases where the tropical countries are willing to take on responsibilities the crediting RL should be below the 'true" BAU baseline. To achieve this aim, it is essential to set up and implement harmonized and/or standardized rules and procedures for the collection, interpretation and consistent processing of various sources of forest data. These include earth observation data [10,11] as well as socio-economic data on the basic drivers and pressures for deforestation at national and international levels.

Data may include historical deforestation area measurements, estimates of the associated emissions and their uncertainties, current forest carbon stocks and carbon stock-change maps partitioned by the various carbon and nitrogen pools (e.g. soil, litter), and forest stand structure (e.g. species, age structure). These data can be sourced from a multitude of independent remote sensing instruments and their derived products such as http://www.geo-wiki.org[12], as well as from *in situ *data (primarily forest inventories) and possibly biophysical ecosystem models. What is important is that the data used by different countries should be publicly known, and models should be applied in a consistent manner by those countries, according to specific data and interoperability standards as well as to the respective greenhouse gas (GHG) accounting rules (e.g. Intergovernmental Panel on Climate Change and the Global Earth Observation System of Systems). The modelling tools themselves should also be standardized and certified.

Other types of input, necessary for countries to undertake consistent development of reference scenarios and planning of REDD policies, include activity data relating to the respective pressures and drivers of deforestation, as well as information on forest management planning, forestry supervision and inspection. Depending on the overall policy context of REDD implementation, such information should *inter alia *include not only forest ownership information, forest management plans with associated annual allowable cuts (AACs), and forest protection, but also information such as transportation infrastructure development, agricultural management data and food consumption projections. Such country-specific driver and governance information would have to be in line with scenarios of environmental and social change in a globally consistent manner. Consequently, emission pathways would also be generated in a consistent manner, addressing the issue of international leakage and possible GHG leakage to other sectors. An example would be land use related leakage in terms of N_2_O emissions to the agricultural sector where REDD constraints land expansion, which must be compensated by intensified cultivation.

Both earth observation data and deforestation driver information could either be collected by national constituencies, according to a negotiated standard, or by international agencies in cooperation with national entities. In many countries, substantial capacity-building efforts would have to be undertaken to provide this information according to globally applicable standards, with sufficient quality and in a geographically explicit manner - if possible. Most importantly, earth observation data on past and the current state of the forest as well as DD driver information have to be collected, reported and subsequently processed in a globally consistent manner.

**Figure 1:**
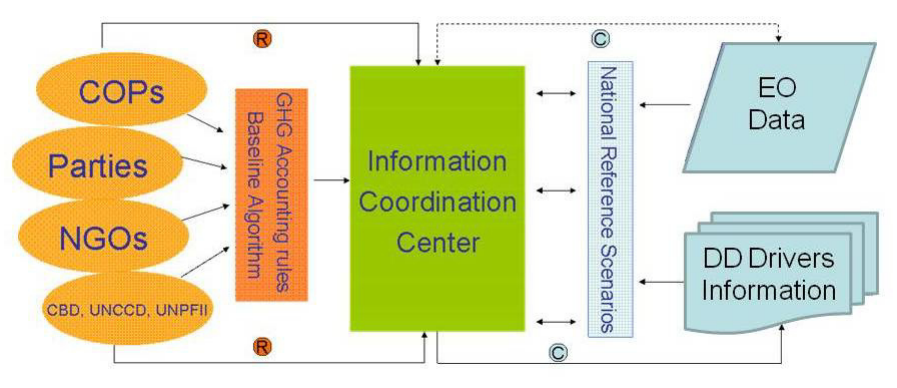
**Institutional set up for determining harmonized reference emission scenarios for REDD**. Under the proposed system national governments would collaborate with the International Emission Reference Scenario Coordination Centre (IERSCC) to build national capacity and to collect relevant deforestation driver and earth observation information. The IERSCC will assist countries in developing rules for establishing globally consistent national reference levels. Relevant entities such as the Conference of the Parties (COPs) to the UNFCCC, non-governmental organisations (NGOs) and other linked international treaties can support and guardrail this process. (EO = Earth Observation, DD = Deforestation and Degradation, R = Rules, C = Consistency).

To achieve this globally consistent use of data and models, and thereby to arrive at fair and efficient REDD reference scenarios, a specific international institutional entity will be needed for the collection, interpretation and consistent processing of various sources of DD-related information at national and international levels. Figure 1 depicts a possible constellation of stakeholders and associated information flows.

### The International Emission Reference Scenario Coordination Centre

To overcome the mentioned challenges we propose an International Emission Reference Scenario Coordination Centre (IERSCC) to assist countries in developing internationally recognized and accepted reference levels. It would act as a clearing house for harmonized data use in reference scenario modelling. The IERSCC, hosted by an independent forestry or land resources research institution of international status, will be tasked to develop global integrated assessment model(s) to deliver sector-specific national scenario information (e.g. trade flows, prices, socio-economic development information) to the respective REDD host countries. The latter would use this information as exogenous variables driving their national reference scenario model/algorithms. Ideally, these scenarios would, in turn, be determined by using geographically explicit, economic, bottom-up type models, whose methodologies could be validated by this or another international validation entity. Such international quality assurance would ensure internationally recognizable REDD reference scenarios of a national model(s) by providing confidence and information security to parties.

Our proposed IERSCC would receive inputs from the respective UNFCCC bodies, in the form of agreed GHG accounting rules, as well as rules (possibly in algorithmic form and parameterization) of the computation procedures for globally consistent national reference scenarios. In this way, the IERSCC would function as an independent technical implementation body to the UNFCCC policy process by supporting and validating consistent collection of earth observation and other DD driver data, based on rules defined by the UNFCCC policy process. The body could also be tasked with developing and applying calibration routines of global top-down modelling with national bottom up modelling to generate consistency between the two.

National-level emission reference scenarios should, as far as possible, be based on geographically explicit data and analysis and allow for down-scaling of national scenarios to assist regional or project level activities. The latter would allow for tailored and targeted national and international REDD actions, since the quantity and the location of deforestation drivers would be better understood [6]. Furthermore, consistent RL setting will be essential for the economic efficiency of international REDD-related compensation mechanisms. Finally, the IERSCC could help in building capacity for REDD response strategies through additional scenario analysis and training of national experts on these issues. It will enable them to conduct their own analysis on national implementation strategies, but also to use part of the IERSCC's data to inform their negotiation strategies in a global context. Furthermore, the IERSCC model results could be used on the individual national level for many other land-use related planning purposes ranging from the analysis of agricultural development strategies all the way to infrastructure planning.

## A REDD finance mechanism ensuring economic and environmental integrity

In the following section we introduce a novel financing approach for REDD, which is able to use the advantages of current proposals while minimizing their disadvantages.

Additionally we specify its functioning under an auctioning mechanism. We will show that this innovative approach optionally allows maximizing social and biodiversity co-benefits (in the following referred to as sustainability co-benefits) while ensuring economic efficiency.

### Combining fund and market strength under an 'International Investment Reserve'

While the risks of direct carbon market inclusion of REDD credits are widely acknowledged, a fund approach might run short of the necessary financing to significantly reduce deforestation. Market-linked approaches like the TDERM [13] and Dual Markets approach [14] do not solve this dilemma, since they provide little incentives for Annex-1 governments to commit to ambitious REDD targets (and thus costs) besides their fossil fuel targets. In the light of current financial constraints on public spending due to the economic crisis and at the same time the overwhelming financing need for climate change adaptation and technology transfer, funds - no matter if originating from AAU earmarking, taxes or other sources- will be constrained.

An interesting alternative is provided by a so-called 'International Investment Reserve' (IIR) for REDD, based on the idea of a "Carbon Federal Reserve" (CFR) [15]. Under the IIR approach REDD providers (developing country governments and/or private carbon projects) would sell their, yet to be created, REDD units to the IIR at an agreed price. The REDD unit price should be below the carbon market credit value and possibly be discounted due to implementation risk and measurement uncertainties [16,17]. The IIR would be financed and managed by Annex-1 governments and possibly private investors. Contributions to the IIR could either be on a voluntary or mandatory basis. Participation for investments would be driven by the economic attractiveness of this scheme. Basically the IIR serves as investment bank for REDD, in which investors provide finance to buy REDD units. These units are then verified and banked, until market conditions are favorable to resell them as fungible MRV-based REDD credits to the carbon market. Given the long-term global emission reduction requirements many models project rising carbon credit prices [18]. This will allow considerable reselling profits - making the IIR an attractive investment option. To avoid market flooding the reselling can be made conditional, e.g. upon a maximum amount of credits per year and/or sufficient market demand signals to maintain competitive carbon prices. Similar to a stock market the IIR members would have an interest in reselling at high carbon market prices to increase the revenue compared to the buying price and thus to limit the risk of credit devaluation. In this way the banking and reselling conditions under the IIR can also contribute to a regulating effect in favor of price floors and caps in the carbon market. Additionally, to ensure environmental integrity reselling can also be made conditional on the overall allowable GHG concentration in the atmosphere, following a global emission budget approach [19].

The reserve would also provide a clear advantage for industrialized country governments for increasing their emission reduction commitments. Under the current situation governments would have to increase their national abatement targets in advance to avoid the risk of REDD credits flooding the carbon market. However, by doing so they take a high risk of belated or insufficient supply of REDD credits in the future. Given the unfavorable governance and capacity situation in many developing countries to successfully implement REDD [20] this risk is real. As a consequence these governments would have to substitute the lacking REDD supply with other emission abatement options which would increase the overall emission reduction costs considerably. Thus, from a strategic perspective governments would not choose higher abatement targets as dominant strategy for REDD, because the supply uncertainty would leave them in a situation of "first mover disadvantage". Under the IIR approach supply uncertainty would diminish, allowing governments to react to supply dynamics more flexible. Investment risks due to delivery failure would still be possible. However, they could be limited, if REDD unit payments from the IIR are divided into up-front financing and continued payments. In case fewer units are provided than initially offered, the difference is subtracted from the remaining payment obligations. Developing countries would also benefit from the IIR approach, since large financial flows could be generated in a timely manner. To increase their confidence in the mechanism, industrialized countries could commit a certain minimum financing amount for each auctioning period.

This is possible, since the IIR is compatible with nationally-obtained contributions in form of AAU earmarking and tax revenue finance for REDD as proposed by the European Commission [21]. At the same time governments or private investors can use other forms of investment. According to their financial share in the IIR each party (i.e. the respective government or private investor) obtains emission units from REDD suppliers. IIR members could either pool all units in a joint portfolio or differentiate them according to different criteria. For the first option they would be bought and collected in a common REDD unit portfolio. For the second option unit pools could be differentiated, e.g. by environmental and social co-benefit level. The latter option might require separate investment pools, if not all members agree on additional incentive payments for higher standards. These REDD units are then transferred into fungible, adjusted REDD credits to be sold at the international carbon market or used for increasing/meeting domestic abatement targets. The IIR approach would also have the advantage that risks of supply failure of REDD units as well as delivery failure of MRV standards could be minimized by pooling large REDD portfolios over time. By validating REDD units through the IERSCC before they are finally transferred the IIR can serve as quality catalyst for the carbon market or for national compliance.

### Auctioning of MRV REDD units to account for sustainability co-benefits

Under a purely carbon-focused REDD approach, national-, regional- or project-level actions would be tailored to maximize emission reductions. However, aggressive implementation of REDD policies could run into conflict with basic food security issues [22], create social conflict and, under certain conditions, lead to further environmental degradation on a total landscape level [7,23]. Such conflicts can only be avoided if REDD policies are appropriately designed and implemented. In this respect, REDD policies can be treated in a similar manner as biofuels, as both are competing for land resources. Thus, it seems paramount that any action under the international REDD mechanism should simultaneously recognize the different ecological and social co-benefits that forests provide. Funds and hybrid-market approaches can better accompany such co-benefits than markets since they could differentiate rules and criteria for REDD co-benefits without being restricted to the carbon value or by credit buyer preferences. However, such approaches are prone to inefficiency in incentive distribution, because they will most likely operate based on fixed co-benefit premiums instead of individual opportunity costs.

The mechanism outlined below describes how the provision of essential co-benefits of REDD can be made attractive and how at the same time incentives can be allocated in a cost-efficient way using auctioning. For illustrative purposes we discuss here a 'sealed bid second price auction' mechanism, where potential buyers classically submit their price bid in sealed envelopes. The buyer with the highest price offered wins the bid, however, only has to pay the second highest bid price submitted. Since the winning bidder wins the difference between both prices it is a dominant strategy for the bidder to bid her true value in a 'sealed bid second price auction'. In a simple ' open ascending price auction' bidders would not bid their true value, as this would eliminate their profit margin.

In the REDD context a 'sealed bid second price auction' can work in a slightly different way. Here, we define the seller to be the supplier of REDD emission reduction units, whereas the buyer is the IIR. The IIR will initially distribute its available investment into a fixed amount of auctioning tranches. Emission reduction unit sellers will then submit sealed bid proposals with a minimum selling price per REDD unit and its targeted selling quantity to the REDD IIR. The IIR selects the best bidder, who then can sell its proposed quantity to the second-minimum selling price. If the tranche still contains money, the next best bidder can sell to the next lowest conditions. This continues until the finance is exhausted.

This auction can then be repeated in tranches of decreasing finance quantity, until the provided finance portfolio is exhausted or the targeted emission reduction quantity of the IIR is reached. The decreasing tranches provide incentives for REDD bidders to engage early in the auction to be able to sell all offered units and thus supply REDD credits at a price which better reflects their true cost of production.

Such an auctioning approach can ensure that a fixed quantitative REDD supply cap is achieved in a competitive setting. It also avoids excessive producer rents by minimizing a REDD arbitrage gap (this is the difference between the REDD costs and the potential revenue from Annex I emission reduction credit supply). Furthermore, auctioning allows for flexibility in targeting the allocation of supply by geographic or thematic areas [24,25].

Co-benefit dimensions cover thematic areas such as the retention of high conservation value forests and biodiversity and the provision of social benefits such as maintenance of employment or cultural services. What is needed for quantifying co-benefits prior to the auction is the identification (and verification) of the absolute or relative magnitude of these co-benefits. The measurement can be orientated on current, widely applied certification processes, or through other means of measuring and verifying sustainability co-benefits.

Using an auctioning approach there are two main options to account for ancillary benefits in the auctioning mechanism. Co-benefits could either be used as a qualifier criterion or as a criterion for the pricing. The qualifier criterion enables to participate in the financial compensation mechanism by achieving a certain quality standard. Besides the overall qualifier criterion to deliver measured, reported and verified (MRV) REDD units, additional social and environmental standards could be set for all REDD credit providers. If the REDD provider fails to achieve them, he would be excluded from the auction. Alternatively, where the aim is maximizing sustainability co-benefits of emission reductions under REDD, the competitive criterion can be the relative provision of sustainability co-benefits. They can be measured as the quantified and certified amount of ecosystem value points and social value points. It can be calculated according to pre-specified co-benefit assessment rules associated with the fungible REDD unit. Ideally, such a value system would be negotiated under the umbrella of a number of UN conventions and charters. However, since a political consensus on this issue is difficult to achieve, the determination of the assessment rules could alternatively be restricted by the parties involved in the financing of such co-benefits. The relative co-benefit performance would then be translated into a co-benefit factor. Such factor can for example range from 0.5 to 1.5. When provided REDD credits ensure maximum co-benefit maintenance then the offered price for the winning bidder would be increased by the factor 1.5. If the winning bidder only provides the lowest possible co-benefit protection his offered price could be discounted by 0.5.

An alternative approach to use the relative co-benefit performance as criterion for the pricing can be realized. Here, the offered REDD units are distinguished into different tranches according to the provided sustainability co-benefit value points.

After a certain amount of REDD units of the highest tier (determined by a minimum amount of co-benefit value points) has been purchased, the auctioneer lowers the minimum points (and the provided finance) to qualify for the next tier of REDD units in the next auction tranche and collects bids at this lower sustainability co-benefit value level. The IIR continues to lower the points (and finance) until the targeted REDD units are bought or the finance portfolio is exhausted.

For both options where sustainability co-benefits are used for the pricing of REDD credits, the sealed bid second-price auction allows maximizing the total sustainability benefit value of a REDD action. This would shift the incentive structure for REDD policy action designs from the simple maximization of emission avoidance to a more comprehensive approach of both emission-avoidance and ecosystem co-benefit maximization.

**Figure 2:**
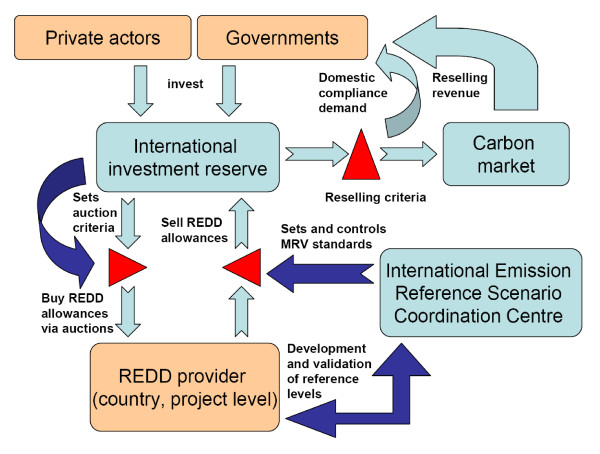
**Institutional relationship**. Relationship among the International Emission Reference Scenario Coordination Centre (IERSCC) the International Investment Reserve (IIR) and the carbon market under an auctioning approach.

The introduced novel design elements of IERSCC for monitoring and RL development and the IIR for auctioning and catalysing REDD credits are interrelated and can help to overcome current methodological and political challenges. Their connections and interdependencies are summarized in Figure 2.

## Conclusion

There is general agreement in REDD policy circles that emission reduction units generated by any REDD mechanism under the UNFCCC must be "real" and environmentally integer. Key to the supply of "real" REDD credits is an appropriate reference level against which additional REDD efforts can be measured, and compensation can subsequently be claimed. For compensation mechanisms, appropriate RL are necessary, irrespective of whether REDD credits are supplied to funds, are made fungible to markets or involve any other REDD implementation design.

As was alluded to earlier, a danger exists that if the methodology for setting RL is not carefully designed it will lead to non additional emission reductions and potentially to an inflated supply of REDD credits. There is the inherent problem of generating and exploiting information asymmetries, which countries will want to exploit by increasing the emission levels for their emission reference scenario. Because the "true" REDD effort is poorly observable, individual market agents are inclined to use the information asymmetry to over-report on their individual efforts and the easiest way to inflate reported efforts is to increase the RL. That is why we have proposed the establishment of an International Emission Reference Scenario Coordination Centre (IERSCC), specifically tasked to establish globally consistent national reference emission scenarios based on standardized and consistent data and algorithms, according to the outcomes of the continuing REDD negotiations under the UNFCCC. RL will need to be established in a globally consistent manner in order to address the problems of geographic and sectoral leakage. The issue of leakage is closely linked to the issue of how drivers of deforestation are included in RL and it rapidly becomes highly contextual. For example, RL of one place or country can depend on the actions of REDD by other market participants. In a particular country where forest conversion is due to the expansion of intensive agriculture, REDD actions in this area can lead to high leakage to other regions/countries. However, if e.g. the driver is extensive cattle-ranching and if REDD measures target the intensification of livestock production systems, then geographic leakage will most likely be small. Thus, RL setting of one country might need to account for the ensemble of global REDD actions, or even more general land-use impacting policies such as biofuel supporting policies.

More realistic reference scenarios would also lead to more transparency and finally to "fairness" in the REDD process. "Real" RL are a precondition for more robust cooperation between parties under the UNFCCC. Asymmetrically inflated RL would lead to windfall profits for the inflating countries, resulting in an unfair allocation of global financial resources dedicated to REDD. Due to its inflated reference emission scenario, country X might receive all of the global REDD resources and country Y would get nothing by imposing a stringent reference scenario and thus loosing competitiveness. However, under a consistent framework of globally harmonized and consistent national reference emission scenarios, countries X and Y would share the globally available REDD resources in a fair manner as they would be compensated for their real efforts.

There are two main problems associated with the asymmetric inflation reference scenario. First, cooperation within tropical countries would be put under threat. Clearly, country Y would try to sabotage negotiations under such conditions, since it would face a loss of revenues from a global REDD mechanism while its competing tropical country would be gaining revenues. Second, REDD credit buyers would face an environmental integrity problem. The entity sourcing REDD credits could *ex-post *be blackmailed for having undermined the environmental integrity of its emission reduction claims and have spent a large share of its resources on REDD hot air.

Besides the prevented information asymmetries, the IERSCC could provide data much cheaper than under pure nationally-based monitoring and earth observation systems. Its associated costs could for example be generated by establishing a tax on sold REDD credits, based on individual donor contributions or by the IIR.

The financing of REDD under an 'International Investment Reserve' would allow the timely provision of large sums for REDD. Sufficient up-front financing can be ensured to tackle deforestation in meaningful quantities, since the IIR allows combining financial resources from national governments targeting market and fund approaches as well as private investments. Details on the management of such IIR still need to be determined, but rich experiences from other study fields exist. The main challenges - similar to the IERSCC - will be to avoid institutional overburdening while at the same time allowing the participation of all relevant stakeholders. The IIR auctioning approach also needs to avoid single country domination such as in the CDM. The participation rules for REDD unit provider should thus take into account their total supply provision to avoid auction domination. For the auctioning mechanism broad participation could be ensured using multiple tranches of finance provision. Subsequent tranches would be lowered in their fixed amount of money to give an incentive to large suppliers for early offers as well as maintaining the option for small (and possibly more expensive) REDD suppliers to participate in the later tranches.

Compliance with environmental and social standards could be ensured through appropriate auditing and possible certification, as an entry condition to participate in the mechanism. Such certification using for example existing REDD standards such as the Climate, Community & Biodiversity Alliance's (CCBA, http://www.climate-standards.org/) could act as a qualifying trait. However, due to heterogeneous priorities and capacities in developing countries, an ambitious standard would most likely overburden the REDD negotiations. Thus, alternatively to a co-benefit qualifying trait a relative quantification of co-benefits as competitive trait provides several advantages. First, it allows maximizing co-benefit provision. But even more important, it can function as additional incentive without excluding REDD providers, which would otherwise fail a high co-benefit standard. We provided alternative options how to incentivize co-benefit performance. The sequential differentiation of tranches according to co-benefit performance would only provide meaningful incentives, if large amounts of REDD units with high co-benefit provision would exist. This however remains speculative. If the co-benefit factor would instead be used as competitive trait, the REDD providers would be incentivized to provide a low unit price and high co-benefit performance without limiting the participation to a specific tranche. The use of the co-benefit factor will also make it harder for REDD providers to speculate on the prices of their co-bidders in each auction, thus limiting the threat of strategic pricing.

What is essential to note is that in such a co-benefit maximising auction design the underlying causes of deforestation and degradation (e.g. poverty) might be attacked more effectively, and would allow for a wide portfolio of REDD implementation instruments. In addition, it needs to be recognized that REDD, if it is purely focused on carbon, will create additional pressure on forests with low carbon stocks such as the Brazilian Cerrado, which is a known biodiversity hotspot. This "biodiversity leakage" effect of REDD within the forest domain needs to be addressed.

To increase the precision of the proposed REDD mechanism with respect to sustainability co-benefit provision, it will be necessary to map with higher precision, and more comprehensively, the ecosystems and societal values *per se*, and agree at the international level on how to quantify co-benefits, such that they could be incorporated into the proposed mechanism. The spatial and temporal deforestation driver data from IERSCC could assist in this task. Besides these more ambitious requirements for the competitive trait, the auction mechanism design should be implemented using robust but simple MRV standards as qualifying criteria to sell REDD units. This is crucial, if sufficient competition should develop, since currently only a handful of countries are "REDD ready". To reach these robust MRV standards REDD readiness funding will be crucial in the coming years. The flexible structure of the IIR will allow early and fluent phasing from such a funding approach towards the proposed investment reserve.

Both Annex I as well as Non-Annex I countries are currently working towards a policy process ensuring that RL are supplied in a transparent, consistent, comparable and accurate manner. It is commonly understood that "real" reference level are a necessary precondition for financial REDD resources to be deployed in a manner that is efficient, effective and, most importantly, distributed among recipients in a fair manner. We argue that an integral and robust REDD policy process ought to be based on independent and globally consistent data compilation, and harmonized computation of appropriate reference scenarios. To achieve this, we advocate the establishment of an 'International Emission Reference Scenario Coordination Centre', as an important part of a robust REDD policy process.

Of similar importance as sound reference levels is the provision of a powerful financing mechanism for REDD, which should not contribute to market flooding but lead to sustained and sustainable investments in forest preservation.

We offer a new financial framework of an 'International Investment Reserve', which enables the timely provision of sufficient financial resources without risking carbon market flooding. The IIR will allow to combine REDD finance generation such as classical market investment and fund approaches. This investment reserve can be used to obtain REDD units from supplier countries under an auctioning approach. This allows to pool risks and uncertainties, and subsequently, to resell these verified credits to the carbon market or use them for national compliance purposes. To avoid market flooding, credits will be banked and the reselling will be constrained by criteria to ensure environmental integrity as well as carbon price floors and caps. This helps to smoothen price volatility.

REDD will not only contribute to mitigating climate change but might also emerge as a major tool to conserve the ecosystem and wider societal values of forests. In this paper, we have explored new ways of how to build "ecosystem and social services" into the carbon economy. Understanding that biodiversity, ecosystem services and other services of forests to society (and the related REDD opportunity costs) are not distributed evenly across the forests of the world, we propose a mechanism design for REDD implementation which allows to maximize carbon and ecosystem co-benefit provision without excluding REDD credit providers.

Consequently, REDD will only be successful in the long term, if it manages the balance between environmental integrity, economic efficiency and political robustness. The REDD mechanism design proposals of two independent REDD institutions, the IERSCC and IIR, introduced in this paper can positively contribute to this endeavour.

## Competing interests

The authors declare that they have no competing interests.

## Authors' contributions

MO had the idea, provided the first draft for the design of the study and critically reviewed the manuscript during each step of production. MH provided the idea for the investment reserve and substantially contributed to several sections of the manuscript, i.e. data analysis and interpretation. FK coordinated the work on this paper and substantially contributed to the analysis and further developing of the ideas. IM provided literature and data and helped drafting paragraphs of this paper. KA, HB, SF, MG, PH, GK, ER and BR provided substantial expert knowledge and ideas during the author's discussions, without which this article could not have been produced. All authors read and approved the final version of the manuscript.
